# Clinical analysis of sinus bradycardia in patients with severe COVID-19 pneumonia

**DOI:** 10.1186/s13054-020-02933-3

**Published:** 2020-05-26

**Authors:** Lijuan Hu, Linjing Gong, Zhilong Jiang, Qibing Wang, Yunzeng Zou, Lei Zhu

**Affiliations:** 1 Department of Pulmonary Medicine, Zhongshan Hospital, Fudan University, 180 Feng Lin Rd., Shanghai, 200032 China; 2 Shanghai Institute of Cardiovascular Diseases, Zhongshan Hospital, Fudan University, 180 Feng Lin Rd., Shanghai, 200032 China

**Keywords:** COVID-19, Severe pneumonia, Sinus bradycardia, Clinical manifestation, ACE2

There were cases of sudden death in some patients infected with COVID-19 including a few of young physicians, which had a huge impact on medical community and society [1]. The unexpected phenomenon lets us think about the underlying problems that caused the sudden death and some issues maybe ignored and that should be appropriately resolved. The initial manifestation of severe COVID-19 pneumonia patients was hypoxemic respiratory failure, accompanied by rapid increased reactive heart rate and susceptibility to supraventricular arrhythmia [2]. It is notable that a proportion of these patients developed sinus bradycardia, which was significantly different from other patients with multiple types of respiratory failure.

In addition to lung injury, cardiac injury has often been reported in patients with COVID-19 [2]. Some experts believed that the virus invasion into myocardium led to severe myocarditis or the severe “cytokine storm”-induced acute myocardial injury may explain the sudden death in some affected patients [3]. It is noteworthy that about 1/3 of the patients with severe illness in our study developed sinus bradycardia (Fig. 1). The troponin and proBNP were basically normal among these patients except for those with renal failure (Table 1). The clinical characteristics of explosive myocarditis and myocardial infarction were not presented among these patients, suggesting these are not the cause of sinus bradycardia in these patients. It was previously reported that no pathological evidence of myocarditis or myocardial microinfarction was observed in the heart of suffered patients [4], consisting with our results. Therefore, we speculated that sudden death among some severe patients with improved symptoms post-treatment may be caused by severe arrhythmia such as ventricular fibrillation induced by severe sinus delay.

**Fig. 1:**
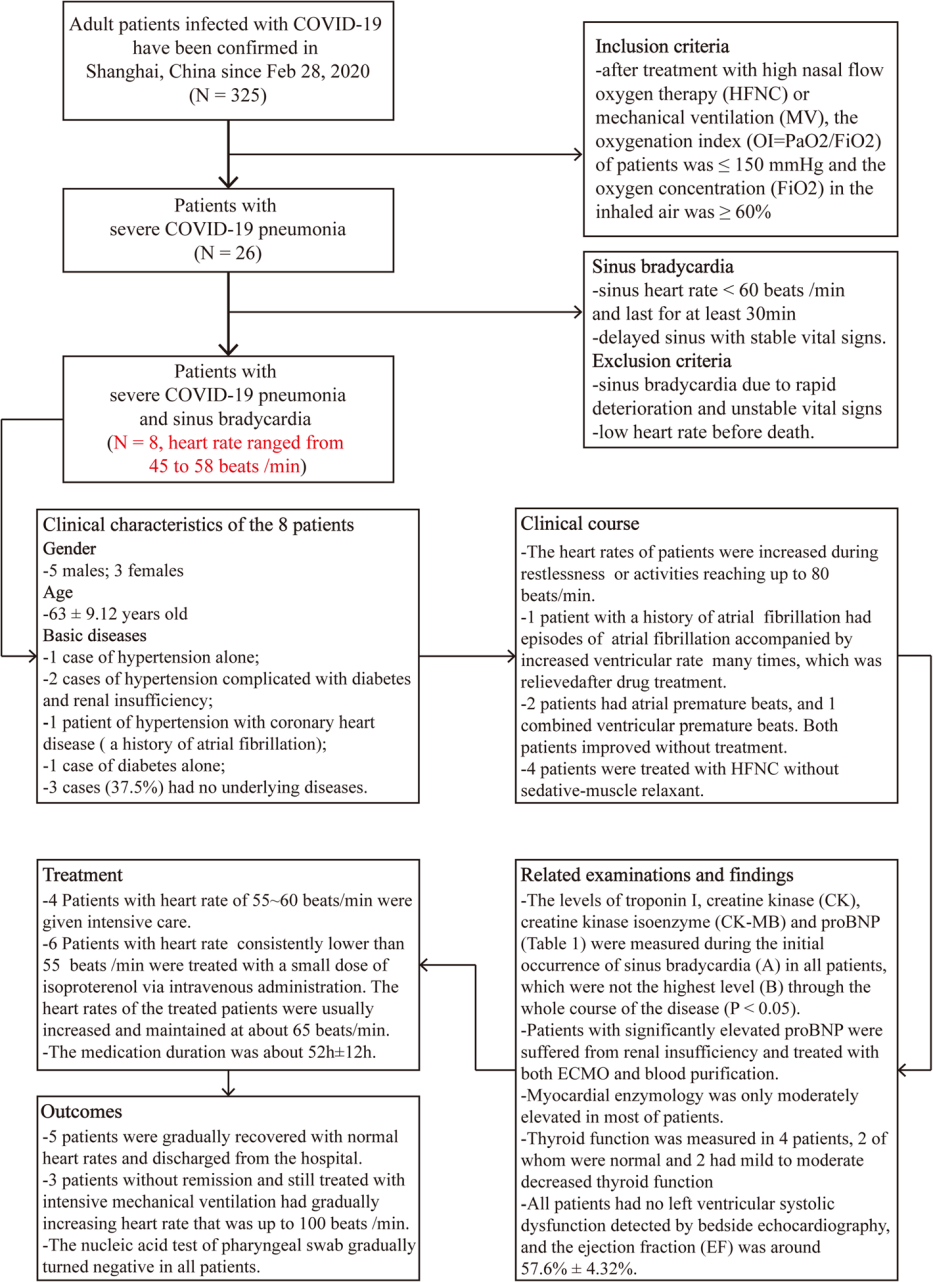
Flowchart of the screening and clinical features of severe COVID-19 pneumonia patients with sinus bradycardia involved in the study

**Table 1:** The related indexes of 8 severe COVID-19 pneumonia patients with sinus bradycardia

Troponin I (ng/ml)		CK (U/L)		CK-MB (U/L)		proBNP (ng/ml)	
A	B	A	B	A	B	A	B
0.01	0.05	416	1154	14	34	128	160
0.02	0.03	30	98	9	11	449	896
0.03	0.15	21	445	8	15	200	354
0.07	0.26	29	76	18	16	909	6312*
0.04	0.23	57	568	12	81	780	4048*
0.02	0.22	34	291	17	28	149	275
0.02	0.03	500	506	19	19	38	38
0.23	0.06	46	568	11	34	449	4048*
Paired *t* tests	1.64	Paired *t* tests	3.39	*Z* value	2.11	*Z* value	2.46
*P*	0.146	*P*	0.012	*P*	0.035	*P*	0.014

Stata 14.0 software was used for the statistical analysis of these data. Paired *t* test or Wilcoxon’s paired rank sum test was used to calculate the corresponding *P* value. Difference is considered statistically significant when *P* < 0.05

*CK* creatine jubase, *CK-MB* creatine kinase isoenzyme MB

*Patients had renal insufficiency and treated with both ECMO and hemodialysis

We found that sinus bradycardia often occurred during sleep. So, deep sleep or sedation may be an important risk factor for sinus bradycardia. A few patients had mild to moderate decreased thyroid function, which was consistent with secondary pathological thyroid syndrome and may also be one of the causes of sinus bradycardia. When viral nucleic acid tests gradually turned negative, the heart rate returned to normal no matter whether the patient’s condition improved or worsened and the uses of catecholamine were gradually discontinued. According to the results, we speculated that the inhibitory effect of virus on sinus node activity was the main cause of sinus bradycardia in these patients.

Previous study indicated that COVID-19 invaded host cells via the receptor angiotensin-converting enzyme 2 (ACE2) [5]. Zou et al. identified specific cell types including myocardial cells which were vulnerable to COVID-19 infection through scRNA-seq data analyses [5]. However, there was no severe myocardial damage or cardiac insufficiency in our patients with sinus bradycardia. We referred the gene ontology (GO) enrichment analysis for ACE2 gene in GeneCards Database (https://www.genecards.org/). Biological processes (BP) for ACE2 gene showed that it not only promoted the contraction of cardiac muscle, but also regulated the cardiac conduction. Donoghue et al. demonstrated that cardiac ACE2 overexpression in transgenic mice caused sudden death in a gene dose-dependent fashion; they also found that increased ACE2 expression led to progressive conduction and rhythm disturbances with lethal ventricular arrhythmias via detailed electrophysiology [6]. In light of those evidences, it may be speculated that the toxic role of virus on cardiac conduction system instead of that generated myocardial damage resulted in a sudden death of patients infected with COVID-19.

Taken together, heart rate monitoring of severe COVID-19 pneumonia patients should be strengthened during treatment, and catecholamines should be appropriately applied when necessary. Moreover, a possible inhibitory influence of the virus on activity of cardiac nervous conduction system including sinus node via ACE2 should not be ignored when studying the pathogenic mechanisms among these patients.

## Data Availability

The data used to support the findings of this study are available from the corresponding author upon request.

## Abbreviations

ACE2: Angiotensin-converting enzyme 2
BP: Biological processes
CK: Creatine kinase
CK-MB: Creatine kinase isoenzyme
EF: Ejection fraction
FiO_2_: Oxygen concentration
GO: Gene ontology
HFNC: High nasal flow oxygen therapy
MV: Mechanical ventilation
OI: Oxygenation index
SARS-CoV: Severe acute respiratory syndrome coronavirus
